# Streamlined Efficient Synthesis and Antioxidant Activity of γ-[Glutamyl]_(*n*≥1)_-tryptophan Peptides by Glutaminase from *Bacillus amyloliquefaciens*

**DOI:** 10.3390/molecules28134944

**Published:** 2023-06-23

**Authors:** Wenjiang He, Xiaoling Huang, Abulimiti Kelimu, Wenzhi Li, Chun Cui

**Affiliations:** 1 Infinitus (China) Co., Ltd., Guangzhou 510640, China; 18688485766@163.com (W.H.); peter.wenzhi.li@infinitus-int.com (W.L.); 2 School of Food Science and Engineering, South China University of Technology, Guangzhou 510640, China; 202121027725@mail.scut.edu.cn; 3 College of Food Science and Pharmacy, Xinjiang Agricultural University, Nongda East Road 311, Urumqi 830052, China

**Keywords:** glutaminase, glutamine, tryptophan, emzymatic synthsis, γ-[glutamyl] _(*n*=1, 2, 3)_-tryptophan peptides, transpeptidation, antioxidant activities

## Abstract

As a group of naturally occurring peptides in various foods, γ-glutamyl peptides possess a unique Kokumi taste and health benefits. However, few studies have focused on the functionality of γ-glutamyl peptides. In this study, the γ-[glutamyl] _(*n*=1, 2, 3)_-tryptophan peptides were synthesized from a solution of glutamine (Gln) and tryptophan (Trp) employing L-glutaminase from *Bacillus amyloliquefaciens*. Four different γ-glutamyl peptides were identified from the reaction mixture by UPLC-Q-TOF-MS/MS. Under optimal conditions of pH 10, 37 °C, 3 h, 0.1 mol/L Gln: 0.1 mol/L Trp = 1:3, and glutaminase at 0.1% (*m*/*v*), the yields of γ-l-glutamyl-l-tryptophan (γ-EW), γ-l-glutamyl-γ-l-glutamyl-l-tryptophan (γ-EEW) and γ-l-glutamyl-γ-l-glutamyl-γ-l-glutamyl-l-tryptophan (γ-EEEW) were 51.02%, 26.12% and 1.91% respectively. The antioxidant properties of the reaction mixture and the two peptides (γ-EW, γ-EEW) identified from the reaction media were further compared. Results showed that γ-EW exhibited the highest DPPH^•^, ABTS^•+^ and O_2_^•−^-scavenging activity (EC_50_ = 0.2999 mg/mL, 67.6597 μg/mL and 5.99 mg/mL, respectively) and reducing power (EC_50_ = 4.61 mg/mL), while γ-EEW demonstrated the highest iron-chelating activity (76.22%). Thus, the synthesized mixture may be used as a potential source of antioxidant peptides for food and nutraceutical applications.

## 1. Introduction

The human body constantly generates free radicals from its various biochemical processes [1]. A moderate amount of radicals plays a beneficial role and can promote cell proliferation and differentiation. However, the excessive accumulation of these reactive species causes varying degrees of damage to biomolecules such as proteins and DNA due to the radicals’ strong oxidizing properties [2], thereby leading to a variety of pathological changes including atherosclerosis, neurodegenerative diseases, age-related degeneration, and cancer at the cell and tissue levels [3,4]. Therefore, maintaining the homeostasis of free radicals in the body has become a key factor in preventing diseases induced by free radicals.

In this context, various free-radical scavenging agents, including antioxidant enzymes, synthetic antioxidants, and natural antioxidants have been developed [5]. In recent years, multifunctional natural antioxidant peptides (food-derived or synthetic peptides) as an alternative to synthetic ones have increasingly attracted the attention of the scientific community because of their desirable antioxidant properties, such as scavenging various free radicals, binding metal ion catalysts, or chelating pro-oxidative transition metals [6,7]. Among these peptides, γ-glutamyl peptides, widely distributed from microorganisms to mammals and detected in a range of dietary sources [8], have become a focus of research due to their high stability in vivo [9], unique Kokumi taste, and health advantages, including antioxidant, anti-inflammatory, hypoglycemic, and intestinal-regulating activities [10]. Accordingly, some γ-glutamyl peptides have been successfully synthesized by γ-glutamyl transpeptidase [11] and l-glutaminase [12]. However, the widespread application of these peptides is limited, as their large-scale and cost-effective production remains challenging.

**Figure 1:**
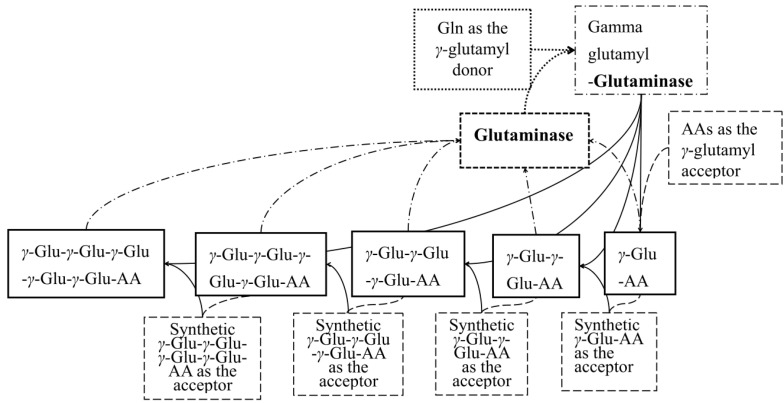
The scheme of synthesis of γ-glutamyl peptides by glutaminase.

As an efficient, cost-effective, and suitable means for large-scale production, enzymatic synthesis finds great application in the preparation of γ-glutamyl peptides. The most common enzyme involved in this process is γ-glutamyl transferase (GGT, EC 2.3.2.2) [10], which can catalyze the transfer of γ-glutamyl moieties from γ-glutamyl compounds to peptides or free amino acids. As a hydrolase, glutaminase mainly hydrolyzes glutamine into glutamic acid. Some microbial L-glutaminase (EC 3.5.1.2) from *Bacillus amyloliquefaciens* and *Aspergillus oryzae*, however, has also exhibited γ-glutamyl transferase activity [12,13], as well as a broader substrate specificity for various acceptors (Figure 1) [13]. Therefore, l-glutaminase has attracted considerable attention owing to its commercial importance in both the pharmaceutical and food industries [14]. Currently, l-glutaminase has been applied successfully in the synthesis of a wide variety of γ-glutamyl compounds, including kokumi γ-glutamyl peptides and bioactive peptides such as γ-glutamyl-cysteine [15], theanine [16], γ-glutamyl-methionine [17] and γ-glutamyl-tryptophan [18,19]. Nevertheless, due to the broader substrate specificity of l-glutaminase, the synthesis mechanism of γ-glutamyl peptide derivatives is much more complicated and still far from entirely understood.

In our previous studies, we have successfully synthesized a peptide mixture including γ-GPs (γ-[Glu]_(*n*=1, 2, 3, 4, 5)_-Phe, γ-[Glu]_(*n*=1, 2, 3)_-Met, γ-[Glu]_(*n* =1, 2, 3)_ –Tyr, γ-[Glu]_(*n*=1, 2, 3)_–Val and γ-[Glu]_(*n*=1, 2, 3, 4)_-Cys) by glutaminase from *Bacillus amyloliquefaciens*, which, we believe, could be applied as antioxidants, a flavor enhancer, and a potential therapeutic drug substitute [15,20,21]. Another important γ-glutamyl compound that has attracted attention is γ-glutamyl-tryptophan. Reportedly, γ-glutamyl-tryptophan has functionalities of anti-tuberculosis action, thymic and splenic cell proliferation, tumor suppression, chemoradiation therapy-induced oral mucositis mitigation, etc. [22,23]. However, there is still no systematic study on the synthesis of γ-glutamyl-tryptophan peptide and its derivatives (di-, tri-, tetra-, and pentapeptides) by glutaminase. Moreover, research on γ-GPs mainly focused on kokumi imparting properties, and few studies have evaluated the health benefits of γ-GPs [18]. Understanding the role of γ-GPs in the antioxidant processes may bring about a new perspective on the application of these peptides, not only on taste enhancement of food but also on pharmaceuticals.

Therefore, one objective of this study was to optimize the synthesis parameters of a series of novel γ-[glutamyl]_(*n*=1, 2, 3, 4)_-tryptophan peptides by glutaminase from *Bacillus amyloliquefaciens*. Another objective was to evaluate the antioxidant activity (DPPH^•^, ABTS^•+^ and O_2_^•−^) scavenging activity, Fe^2+^-chelating ability, and power-reducing assays) of the resultant reaction medium and two main peptides.

## 2. Results and Discussion

### 2.1. Acceptor Amino Acid Screening

Our preliminary study showed that the type of substrate amino acids has a significant impact on peptide synthesis. Therefore, we thought that it was necessary to screen out a suitable amino acid for peptide synthesis with a high yield. For this, a tentative synthesis of γ-Glu-peptides employing Gln and 18 other amino acids at 37 °C for 3 h was performed, and the antioxidant properties (DPPH^•^-scavenging activity, Fe^2+^ chelating ability and superoxide anion scavenging activity) of the mixture before and after enzymatic synthesis were evaluated. Interestingly, the antioxidant activity of the peptides mixture prepared from Gln-Cys showed the highest increase, followed by Gln-Trp upon enzymatic treatment. The synthesis and antioxidant activity of γ-Glu-peptides from the mixture of Gln-Cys has been previously discussed [15]. We considered that the synthesis and functionality of γ-Glu-peptides from the mixture of Gln-Trp should be studied further.

### 2.2. Identification of γ-[Glu]_n_-Trp Peptides

**Figure 2:**
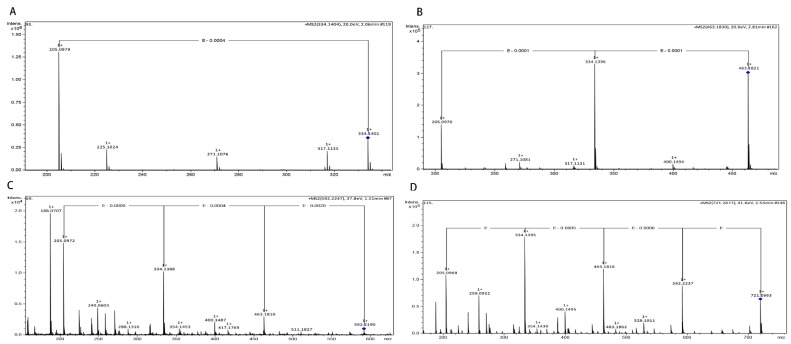
Mass spectra of products in the post-enzymatic reaction mixture with Gln and Trp as the substrates: (**A**) γ-l-glutamyl-l-tryptophan (γ-EW); (**B**) γ-l-glutamyl-γ-l-glutamyl-l-tryptophan (γ-EEW); (**C**) γ-l-glutamyl-γ-l-glutamyl-γ-l-glutamyl-l-tryptophan (γ-EEEW); (**D**) γ-l-glutamyl-γ-l-glutamyl-γ-l-glutamyl-γ-l-glutamyl-l-tryptophan (γ-EEEEW). (The blue squares in the figure represent the peak position of the target product).

**Figure 3:**
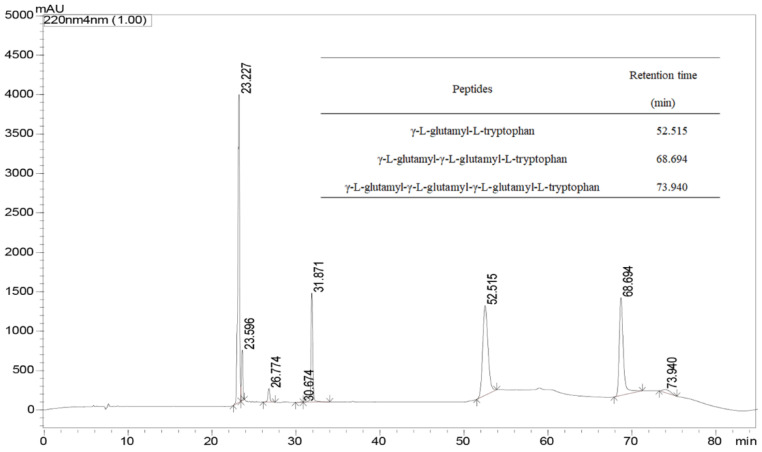
An HPLC chromatogram (λ = 220 nm) of the post-enzymatic reaction mixture containing γ-[Glu]_(*n*=1, 2, 3)_-Trp. Inserted is the table showing the retention times of γ-[Glu]_(*n*=1, 2, 3)_-Trp.

The previously prepared γ-[Glu]*_n_*-Trp peptides utilizing Gln and Trp as the substrates were subjected to instrumental analyses by UPLC. Based on the molecular mass and fragment information from the UPLC-Q-TOF-MS/MS analysis, four γ-l-glutamyl-peptides including γ-l-glutamyl-l-tryptophan (γ-EW), γ-l-glutamyl-γ-l-glutamyl-l-tryptophan (γ-EEW), γ-l-glutamyl-γ-l-glutamyl-γ-l-glutamyl-l-tryptophan (γ-EEEW) and γ-l-glutamyl-γ-l-glutamyl-γ-l-glutamyl-γ-l-glutamyl-l-tryptophan (γ-EEEEW) were identified in the reaction mixture (Figure 2). However, only 3 γ-glutamyl peptides were detected under the experimental conditions, and γ-EEEEW was not detected (Figure 3), possibly because the low yield of γ-EEEEW did not reach the detection limit of HPLC, which also indicates that the UPLC-MS/MS method is more sensitive than the HPLC method. Thus, these results clearly indicate that γ-Glu-peptides have been successfully synthesized by the l-glutaminase of *Bacillus amyloliquefaciens* employing L-Gln and L-Trp as the substrates.

### 2.3. Parameter Optimization of γ-Glu-Peptides Synthesis

#### 2.3.1. Effect of pH

**Figure 4:**
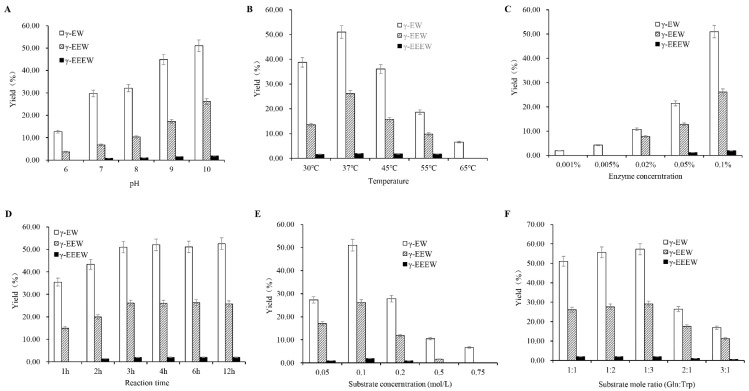
The yield (%) of the γ-[Glu]_(*n*=1, 2, 3)_-Trp peptides obtained under various reaction conditions: (**A**) pH, (**B**) temperature, (**C**) enzyme load, (**D**) synthesis time, (**E**) substrate concentration, (**F**) donor/acceptor ratio.

Reaction pH is a crucial factor in enzymatic synthesis. Substrates should be nonionized for kinetically controlled peptide synthesis to occur. The variation in pH affects the ionization equilibrium (pKa value) of the amino substrates, which finally influences the peptide synthesis [24]. The synthesis of γ-glutamyl peptides by glutaminase has been known to proceed in two steps: the formation of γ-glutamyl-enzyme intermediate and the transfer of the γ-glutamyl group to an acceptor (amino acid or peptide) or water (hydrolysis reaction) [10]. To find out the optimum pH for γ-glutamyl peptides synthesis, we investigated the influence of different pH. The total concentrations of all monitored γ-glutamyl peptides in the reaction media are presented in Figure 4A. The pH of the reaction mixture has a large impact on the yield of γ-glutamyl compounds. Glutaminase displayed some activity under alkaline to neutral conditions, and the activity kept increasing until pH 10.0, which is similar to our previous results [15]. At pH 6, only 2 different peptides, γ-EW and γ-EEW, were detected, whereas 3 different peptides, γ-EW, γ-EEW, and γ-EEEW, were detected in the pH range of 7–10. The differences in γ-glutamyl compounds observed in the reaction mixture at different pH could be attributed to the effects of pH variation on the active site of the enzyme and the ionic state of the substrate. Besides, the broader substrate specificity of glutaminase for various acceptors is also associated with peptide diversity in the reaction medium [17]. According to the UPLC-Q-TOF-MS/MS-analysis results above, it is also possible that pentapeptide formed via the enzymatic processes was not detected in our experiment. The most abundant peptide was γ-EW, followed by γ-EEW and γ-EEEW, which was possibly the result of the preferential affinity of the enzyme to amino acid or low molecular peptides [21]. An increase in pH from 6−10 led to an increase in the conversion yield of peptides, and the yield of all γ-glutamyl-peptides showed the highest value at pH 10, resulting in 51.02%, 26.12%, and 1.91% yield for γ-EW, γ-EEW, and γ-EEEW, respectively. The conversion rate was much higher than that of our previous study on γ-glutamyl peptide synthesis from the mixture of Gln and Cys [15]. These results indicate that glutaminase exhibited a broader range of pH to efficiently synthesize γ-glutamyl peptides.

#### 2.3.2. Effect of Temperature

Temperature is another critical factor influencing the yield of enzymatic reactions. An increase in temperature leads to an increase in the thermal motion of the substrate, thus increasing the collision efficiency between the substrates and enzymes. Moreover, the activity of the enzyme would be reduced due to the denaturation at a high temperature. The optimum temperature for the synthesis of γ-glutamyl peptides by glutaminase was investigated at ranges between 20–65 °C (Figure 4B). When the syntheses were conducted between 30–55 °C, three peptides, namely γ-EW, γ-EEW and γ-EEEW, were detected, and the conversion yield of all three γ-glutamyl peptides increased remarkably with the increasing reaction temperature from 30–37 °C, and then decreased significantly until 55 °C. At 37 °C, the enzyme showed the highest activity and a further increasing reaction temperature to 65 °C; only the γ-glutamyl dipeptide with the lowest yield was detected in the reaction media. The optimal reaction temperature reported here falls into the range of the optimum temperature observed in our previous research [15] and other studies [23].

#### 2.3.3. Effect of Enzyme Load

The enzyme concentration utilized in synthesis has the largest impact on the cost of peptide production; therefore, a minimization of the amount of glutaminase addition is critical. Consequently, the effect of enzyme concentration on the conversion reaction was studied to determine the proper amount of enzyme required for the maximum formation of the product. The effect of enzyme (0.001–0.1%) concentration on the synthesis of γ-glutamyl peptides was investigated at four different enzyme concentrations while the other parameters were kept constant. As shown in Figure 4C, the enzyme concentration had a greater impact on the yield of γ-glutamyl peptides, and an increase in enzyme concentration led to a significant increase in the yield of γ-glutamyl peptides. Here, the concentration of the yield of γ-EW, γ-EEW, and γ-EEEW increased significantly when the enzyme concentration was increased from 0.05% to 0.1%. At lower enzyme concentrations (0.001–0.005%), even though the peptide yield increased significantly with increasing enzyme concentration, only dipeptide was detected in the reaction mixture. This variation in the γ-glutamyl peptide composition with increasing enzyme concentration may be due to the increased binding of the enzymes with the peptides, thereby producing γ-glutamyl peptides of high molecular weight by the transpeptidation reaction [25].

#### 2.3.4. Effect of Synthesis Time

The synthesis time is one of the most crucial production parameters during enzymatic synthesis. For the successful synthesis of γ-glutamyl peptides, it is expected that both the amino acid and the subsequently synthesized γ-glutamyl peptides must be adequately contacted with the enzyme because insufficient enzymatic synthesis time causes the substrate not to be completely transformed into γ-glutamyl peptides. Considering this, the yield of peptides in enzymatic reaction over time was obtained, and the results are shown in Figure 4D. Within the first-hour reaction, only γ-EW and γ-EEW were detected in the sample, and all three peptides appeared after that. The yield of peptides was increased gradually within 3 h of reaction and almost reached the highest yield after 3 h of incubation. Thereupon no significant difference in peptide yield occurred, indicating that the reaction had reached equilibrium after 3 h of synthesis. This is in line with our previous study results for γ-[Glu]_(*n*=1, 2, 3, 4)_ -Cys peptides synthesis [15].

#### 2.3.5. Effect of Substrate Concentration

As illustrated in Figure 4E, substrate concentration had a significant effect on the production yield and composition of the reaction medium. An increase in substrate concentration from 0.05–0.1 mol/L led to a significant increase in γ-EW, γ-EEW and γ-EEEW yield from 27.30%, 17.07% and 0.93% to 51.02%, 26.12% and 1.91%, respectively. A further increase in the substrate concentration, however, led to a decrease in the peptide yield. When the substrate concentration increased to 0.75 mol/L, only γ-EW was observed with a yield of 6.74% in the reaction medium, indicating that the excessive substrate may lead to the formation of small peptides due to the preferential binding (lower K_m_) of enzymes to free amino acids instead of di- or tri-peptides [15]. According to reaction equilibrium, enzymatic reaction yield is affected significantly by substrate concentration. Usually, a higher substrate concentration gives rise to a higher peptide yield until the enzymes reach their saturated binding. After that, a further increase in substrate concentration has no obvious effect on the yield. In our experiment, the peptide yield was calculated relative to the concentration of glutamine. At a constant product concentration, the concentration of glutamine increased, so the yield showed a relative decline.

#### 2.3.6. Effects of Donor/Acceptor Ratio

For enzymatic synthesis, the donor to acceptor ratio is regarded as a critically important parameter, and this method is frequently used to increase production. The influence of the donor/acceptor molar ratio on the formation of γ-glutamyl peptides was studied in the presence of variable amounts of one reactant over another. The relationship between the synthesis efficiency of γ-glutamyl peptides and the molar ratios of donor and acceptor is shown in Figure 4F. With an increasing concentration of Trp when Gln was kept constant, it was observed that only the γ-glutamyl dipeptide yield increased slightly, while the yield of other peptides kept almost constant. This behavior is interpretable based on the enzyme kinetic. In the transpeptidation reaction, the transfer of the γ-glutamyl moiety from the intermediate γ-glutamyl-enzyme to the acceptor is the rate-limiting step, and the formation of γ-glutamyl-enzyme is faster. An excessive amount of Trp, therefore, does not considerably affect product formation. Subsequently, the effect of donor concentration (Gln) on peptide production was investigated. Increasing the quantity of the donor gives a significantly decreased yield of the product, which is also associated with the γ-glutamyl dipeptide-synthesis mechanism [18]. At the beginning of the reaction, the roles of Gln as the γ-glutamyl donor and Trp as the γ-glutamyl acceptor are distinguishable. However, when the concentration of the produced γ-glutamyl-peptide has reached a certain value, it becomes an γ-glutamyl donor, thereby facilitating the subsequent transpeptidation reaction [10].

### 2.4. Antioxidant Activity

The resulting mixture was lyophilized and the antioxidant activity of the synthetic mixture and its main components, γ-EW and γ-EEW, was evaluated for antioxidative activity by utilizing various antioxidant assays.

#### 2.4.1. DPPH^•^ Scavenging Assay

**Figure 5:**
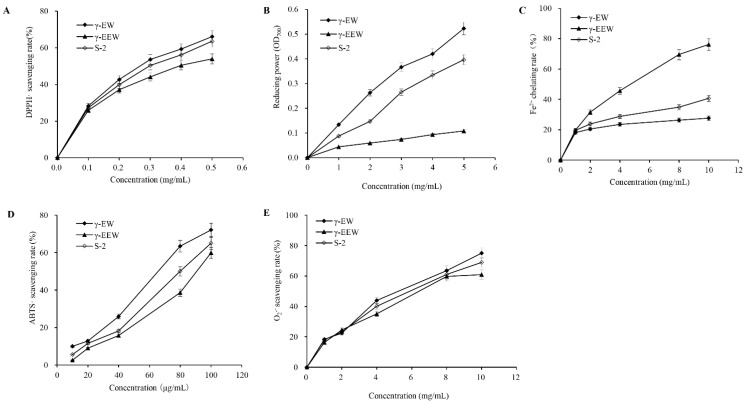
The antioxidant activities of γ-l-glutamyl-l-tryptophan (γ-EW), γ-l-glutamyl-γ-l-glutamyl-l-tryptophan (γ-EEW), and Gln-Trp mixture (S-2) evaluated as the (**A**) DPPH-scavenging activity, (**B**) ABTS-scavenging activity, (**C**) Reducing power, (**D**) Ferrous ion-chelating activity, (**E**) Superoxide anion scavenging activity.

Having established strategies for γ-glutamyl-peptides synthesis, we proceeded to further test the antioxidant ability of γ-EW, γ-EEW, and S-2 (synthesized mixture sample). DPPH^•^ is a stable free radical widely used to investigate the scavenging activity of natural compounds in a non-aqueous medium. Accepting electrons and hydrogen from antioxidants leads to a decrease in absorbance, and the decrease in absorbance at 517 nm is taken as a measure of radical-scavenging activity [26]. The DPPH^•^ radical-scavenging activity of γ-EW, γ-EEW, and S-2 was measured by increasing the concentration from 0 to 0.5 mg/mL (Figure 5A). The results showed that all three samples exhibited a significant dose-dependent (*p* < 0.05) scavenging activity, with γ-EW peptide exerting the highest. The antioxidant activity of a peptide is associated with many factors, among which the amino acid composition is crucial. Containing hydrophobic amino acids including Trp in the peptide sequence was reported to be correlated with DPPH^•^ radical scavenging activity because aromatic and hydrophobic amino acids facilitate the interaction between antioxidants and DPPH^•^ radicals by improving the solubility of peptides in the lipid phase [27]. Moreover, although it has previously been reported that negatively charged acidic amino acid (Glu) contributed to DPPH^•^ radical scavenging activity [28], it was noticed that an increase in Glu residue led to a slight decrease in DPPH^•^ radical scavenging activity, which might be due to their discrepancy in molecular weight. Although there are some contradictory results [29], in accordance with our results, several studies have reported that peptides of low molecular weight presented a higher radical scavenging activity [30,31]. Concerning EC_50_ values, the sequence of DPPH^•^ scavenging activity was γ-EW (0.2999 mg/mL) > S-2 (0.3288 mg/mL) >γ-EEW (0.4109 mg/mL). Sample S-2 showed intermediate DPPH^•^ scavenging activity. This moderate DPPH^•^ scavenging activity of sample S-2 should be caused by the combined effects of free amino acids and peptides existing in the reaction medium.

#### 2.4.2. ABTS^•+^ Radical-Scavenging Activity

Antioxidants exert their radical-scavenging ability via various mechanisms. Therefore, it is necessary to employ different evaluation methods to fully understand the antioxidant activity of a substance. The blue/green radical ABTS^•+^, generated by oxidation of ABTS^•+^ with potassium persulfate, is employed to determine the scavenging activities of both lipophilic and hydrophilic compounds. The mechanism of this assay is based on the ability of antioxidants to donate a hydrogen atom or an electron to quench the ABTS^•+^ radicals [32]. In the range of concentration tested, the ABTS^•+^ radical-scavenging activity of all three samples increased significantly (*p* < 0.05) with increasing concentration, and γ-EW exhibited the highest radical-scavenging ability, followed by S-2 and γ-EEW (Figure 5B). These results are in accordance with other reports, where it has been stated that the ABTS^•+^ radical scavenging activity of a peptide was the result of Tyr, Trp, and Cys residues in peptide sequence and the difference in molecular weight [33]. Like the exhibited DPPH^•^ radical scavenging activity, intermediate ABTS^•+^ radical-scavenging activity was observed for sample S-2. The EC_50_ value of S-2 (79.8677 μg/mL) was lower than that of γ-EEW (91.0820 μg/mL) and higher than that of γ-EW (67.6597 μg/mL), indicating that all three samples inhibited a higher ABTS^•+^ radical at low concentrations, a result superior to those of some reported antioxidant peptides, such as cottonseed peptides [30] and elastin peptide [34].

#### 2.4.3. Reducing Power

The reducing-power assay is often used to quantify the ability of an antioxidant to neutralize reactive species via donating electrons [35] and may serve as a significant indicator of the antioxidant activity of a compound. As shown in Figure 5C, in the investigated concentration range, the reducing power of γ-EW and S-2 exhibited a significant (*p* < 0.01) increase with increasing concentration, while a slight increase in reducing power was observed for γ-EEW. This increase in reducing power for γ-EW can be attributed to indolic groups of tryptophan playing roles as hydrogen donors [36]. Moreover, the increase in the Glu residue in the peptide sequence led to a significant decrease in the reducing power, indicating that Glu was negatively correlated with reducing power. Moreover, the EC_50_ values for γ-EW, γ-EEW, and S-2 were 4.61 mg/mL, 24.23 mg/mL, and 6.13 mg/mL, respectively, indicating that γ-EW peptide was the main ingredient that exerted reducing-power activity.

#### 2.4.4. Ferrous Ion-Chelating Activity

As one of the essential micronutrients, iron has various biological functions in organisms. However, an excessive amount of ferrous ion participates in various oxidation reactions and converts hydrogen peroxide to hydroxyl radicals via the Fenton reaction [37]. Therefore, iron-chelating activity is also one of the important properties of antioxidants. As shown in Figure 5D, the iron-chelating activity of γ-EEW increased rapidly with increasing concentrations, while no significant changes in iron-chelating activity were observed for γ-EW and S-2 with increasing concentration after 1 mg/mL of concentration. The results indicated that an increase in the Glu residue within the γ-EEW peptide sequence was closely related to its Fe^2+^-chelating activity. In agreement with this, Ref. [38] reported that amino acid residue such as Glu within the sequence of a peptide is crucial to its Fe^2+^-chelating activity because the carboxyl and amino groups in its side chain can bind Fe^2+^. Moreover, based on the results presented, it could be suggested that, to some extent, it is not only the amino acid composition that affects the iron-chelating activity of a peptide, for a large molecular peptide chain might be crucial for the iron chelating ability of a peptide. Even though there are some contradictory results [30], ours are well in line with some studies [39].

#### 2.4.5. Superoxide Radical Scavenging Activity

The superoxide anion radicals (O_2_^•−^) form an important reactive oxygen species relevant to food and biological systems. This species is not only related to the oxidative degradation of lipids but also essential in regulating apoptosis and aging [40]. It is therefore commonly used to determine the superoxide anion radicals-scavenging activity of antioxidants. The superoxide radical-scavenging activity of γ-EW, γ-EEW, and S-2 was determined at 0−10.0 mg/mL. As shown in Figure 5E, the activity had a concentration-reliant trend for all samples within 8 mg/mL, and the superoxide radical-scavenging activity of γ-EEW reached a plateau afterwards, while the activity of the other two samples increased linearly with increasing concentration. Even though the γ-EW showed the highest activity, followed by S-2 and γ-EEW, the differences in EC_50_ were insignificant (γ-EW: 5.99 mg/mL, S-2: 6.31 mg/mL, γ-EEW: 6.45 mg/mL).

## 3. Materials and Methods

### 3.1. Materials

Commercial γ-[Glu]_(*n*=1, 2, 3, 4)_-Trp were purchased from Peptide Biological Technology Co., Ltd. (Nanjing, China). Commercial amino acids were purchased from CapitalBio Corporation (Shanghai, China). L-glutaminase from *Bacillus amyloliquefaciens* was purchased from Amano Enzyme China Ltd. (Shanghai, China). Acetonitrile and formic acid were of HPLC grade, and other solvents and chemicals were of at least analytical grade, and all were purchased from CapitalBio Corporation (Shanghai, China).

### 3.2. γ-Glutamyl Acceptor Amino Acid Screening

For the amino acids screening, first 20 mM Gln and 20 mM amino acids (Ala, Val, Pro, Met, Gly, Ser, Lys, Arg, His, Leu, Ile, Phe, Trp, Cys, Tyr, Asn, Glu, Gln) were mixed in a ratio of 1:1, and the mixture (pH 10) was incubated at 37 °C for 3 h upon addition of 0.1% (*m*/*v*) glutaminase. The reaction was terminated by holding the samples at 90 °C for 10 min. The resultant samples were evaluated for their antioxidant activities (2,2, -Diphenyl-1-picrylhydrazyl (DPPH^•^), scavenging activity, reducing power, Fe^2+^-chelating ability and superoxide anion (O_2_^•−^) scavenging activity).

### 3.3. Identification of γ-[Glu]_(n=1, 2, 3, 4)_-Trp Using UPLC-Q-TOF-MS/MS

The γ-[Glu]_(*n*=1, 2, 3, 4)_-Trp peptides in the samples were analyzed by the UPLC-Q-TOF-MS/MS system [21]. Agilent 1290 series UPLC system (Agilent Technologies, Palo Alto, CA, USA) equipped with an Agilent ZORBAX RRHD SB-C18 column (2.1 mm × 50 mm, 1.8 μm; maintained at 30 °C) was used to separate the peptides. A maXis Impact Q-TOF MS/MS system (Bruker Daltonics, Beijing, China) equipped with an electrospray ionization (ESI) probe was used for detection. Mobile phase A was 0.1% formic acid-methanol water solution, and mobile phase B was 0.1% formic acid-water solution. The flow rate was maintained at 0.5 mL/min throughout the analysis, and the elution conditions were 0–5 min, 90–85% (A); 5–10 min, 85–20% (A); 10–15 min, 20–90% (A); 15–25 min, 90% (A). A 10-μL aliquot of each sample was injected for analysis, and the mass range was from 50 to 1000 *m*/*z*.

### 3.4. Optimization of γ-Glutamyl Peptides Synthesis Conditions

The γ-[Glu]*_n_*-Trp peptides were prepared according to our previous research with slight modification [15]. Single-factor experiments were employed for the optimization of the peptide synthesis, and the reaction parameters included pH (6–10), temperatures (30–65 °C), substrate molar ratios (Gln: Trp = 3:1, 2:1, 1:1, 1:2, 1:3), substrate concentration (0.05–0.75 mM), enzyme concentration (0.001–0.1% m/v), and time (1–12 h). The γ-[Glu]*_n_*-Trp peptides in the samples were measured by UPLC, and their yields were calculated as follows:

(1) $$\mathrm{Yield}=\frac{M_{1}}{M_{0}}\times 100\%$$

where: *M*_1_ is the amount of γ-[Glu]*_n_*-Trp peptide and *M*_0_ denotes the initial amount of Gln.

### 3.5. Determination of Antioxidant Activity

#### 3.5.1. DPPH^•^ Scavenging Activity

DPPH^•^-scavenging activity was measured following previous research [41]. The reaction was initiated by the addition of 2 mL of 0.2 mM DPPH in ethanol into 2 mL of test samples. After the reaction mixture had been allowed to stand for 30 min at room temperature, its absorbance at 517 nm was immediately measured using a UV–VIS-NIR spectrophotometer (UV-3600, Shimadzu Co., Kyoto, Japan) [42,43]. The scavenging rate was calculated as follows:

DPPH·scavenging activity (%) = [(A_0_ − A_1_)/A_0_] × 100 (2)

where: A_0_ and A_1_ denote the absorbance values in the absence and presence of the test sample, respectively.

The EC_50_ value was defined as an effective concentration of peptide required to scavenge 50% of the radical activity.

#### 3.5.2. ABTS Radical Scavenging Activity

ABTS radical scavenging ability was measured according to the method of Agrawal with minor modifications [44]. Briefly, 5 mL, 7 mmol/L 2,2′-azinobis(3-ethyl-benzothiazoline-6-sulphonate) (ABTS) solution was mixed with 88 μL, 140 mmol/L potassium persulphate solution and kept in the dark at an ambient temperature for 12–16 h to obtain ABTS+ cation. Then the ABTS stock solution was diluted to an absorbance of 0.7 ± 0.05 at 734 nm with distilled water. As a distilled water control, the free radical-scavenging activity was measured at 734 nm after mixing 20 μL of the sample (0.1, 0.2, 0.3, 0.4, 0.5 mg/mL) with 980 μL of ABTS working solution in dark for 10 min at room temperature.

The ABTS radical-scavenging activity was calculated as follows:

ABTS radical-scavenging activity (%) = OD (Control) − OD (Sample)/OD (Control) × 100(3)

where: OD is absorbance of the control and sample.

#### 3.5.3. Reducing Power

The method described by [41] was used to evaluate the reducing power activity of the samples. The sample solutions (1.0 mL) were added to a mixture of 2.5 mL phosphate buffer (200 mM, pH 6.6) and 2.5 mL K_3_[Fe(CN)_6_] (1%, *w*/*v*). Then, 2.5 mL of trichloroacetic acid (10% *v*/*v*) was added to stop the reaction after keeping the mixture in a water bath (50 °C) for 20 min. Finally, 0.5 mL of FeCl_3_ 0.1% *m*/*v* and 2.5 mL of distilled water were added to 2.5 mL of the resultant mixture, and the absorbance was recorded at 700 nm by a UV–VIS-NIR spectrophotometer (UV-3600, Shimadzu Co., Kyoto, Japan).

#### 3.5.4. Fe^2+^-Chelating Ability

The Fe^2+^-chelating ability was assessed as described by [45]. Briefly, 0.5 mL of the sample was mixed with 0.05 mL FeCl_2_ (2 mM) and 0.2 mL ferrozine (5 mM). After the mixture was kept at 25 °C for 10 min, the absorbance was measured at 562 nm employing a UV–VIS-NIR spectrophotometer (UV-3600, Shimadzu Co., Kyoto, Japan). The Fe^2+^-chelating activity was calculated utilizing the following formula:

Fe^2+^ chelating ability (%) = [(A_0_ − A_1_)/A_0_] × 100 (4)

where: A_0_ denotes the absorbance without a test sample (with distilled water in its place), and A_1_ denotes the absorbance with the test sample.

#### 3.5.5. O_2_^•−^ Scavenging Activity

The O_2_^•−^ scavenging activity was measured according to previous research with slight modifications. Briefly, 1 mL of the test sample, nitrotetrazolium blue chloride (2.25 mM), and NADH (624 mM) were added to 3 mL of Tris-HCl buffer (16 mM, pH 8.2), respectively. The reaction was initiated by adding 1 mL of phenazine methosulphate solution (120 µM) and proceeded at 25 °C for 5 min. The absorbance of the mixture was measured at 560 nm by UV–VIS-NIR spectrophotometer (UV-3600, Shimadzu Co., Kyoto, Japan). The O_2_^•−^-scavenging rate was calculated as follows:

O_2_^•−^ scavenging rate% = [(A_0_ − A_1_)/A_0_] × 100 (5)

where: A_0_ and A_1_ denote the absorbance values without or with a test sample.

### 3.6. Statistical Analysis

Statistical analyses were performed utilizing SPSS 16.0 statistical software. All experiments were effectuated in triplicate, and the results are expressed as the mean ± standard deviation (SD). The difference between mean values was determined by Pair-Sample t-Test at an α-level of 5%.

## 4. Conclusions

In conclusion, γ-[glutamyl]_(*n*=1, 2, 3)_-tryptophan peptides with strong antioxidant activity can be synthesized efficiently from the mixture of glutamine and tryptophan utilizing glutaminase from *Bacillus amyloliquefaciens*. The optimal synthesis conditions were: pH 10, temperature 37 °C, reaction time 3 h, 0.1 mol/L Gln: 0.1 mol/L Trp = 1:3, and glutaminase at 0.1% (*m*/*v*). Under such optimum conditions, yields of 51.02%, 26.12%, and 1.91% were achieved for γ-EW, γ-EEW, and γ-EEW, respectively. Two peptides, γ-EW and γ-EEW, were discovered to be the main antioxidant peptides in the reaction mixture. Amongthese, γ-EW exhibited the highest DPPH^•^, ABTS^•+^ and O_2_^•−^ scavenging activity (EC_50_ = 0.2999 mg/mL, 67.6597 μg/mL and 5.99 mg/mL, respectively) and reducing power (EC_50_ = 4.61 mg/mL), while γ-EEW demonstrated the highest iron-chelating activity (76.22%). The molecular weight and the presence of aromatic (Trp) and acidic (Glu) amino acids were the key determining factors of the antioxidant activity of these peptides. The results suggested that the resultant reaction mixture might be useful for antioxidant food additives, dietary nutrients, and pharmaceutical agents. However, further study of their in vivo antioxidant activities is needed.

## Data Availability

The data presented in this study are available on request from the corresponding author.

## Sample Availability

Samples of the compounds are available from the authors.
